# Primary Raynaud's Phenomenon of the Tongue

**DOI:** 10.7759/cureus.67417

**Published:** 2024-08-21

**Authors:** Rehman Basharat, Jacob Riordan, Ghassan Samara

**Affiliations:** 1 Otolaryngology, Stony Brook University, New York, USA

**Keywords:** tongue pain, raynaud disease, tongue, primary raynaud's, raynaud's phenomena

## Abstract

Raynaud's phenomenon is a condition characterized by intermittent vasoconstriction of arteries in the fingers and skin, triggered by cold temperatures or emotional stress, aimed at conserving body heat. This condition is classified into primary and secondary forms, with secondary Raynaud's often linked to connective tissue diseases, medications, infections, and occupational exposures. A notable clinical case involves a 51-year-old male experiencing episodes of painful, white discoloration of the tongue, which were managed through a comprehensive diagnostic process, including rheumatological and cardiological evaluations, to rule out connective tissue diseases and cardiac dysfunction.

We highlight the complex pathophysiology of Raynaud's, involving vascular, neurogenic, and immune mechanisms. Management strategies focus on lifestyle modifications and pharmacologic treatments, such as calcium channel blockers, to reduce attack frequency and severity. For refractory cases, advanced therapies, including phosphodiesterase inhibitors, intravenous prostaglandins, and surgical sympathectomy, may be considered. Effective diagnosis and individualized treatment are crucial for preventing complications and improving patient outcomes.

## Introduction

Raynaud’s phenomenon is a complicated condition where the arteries in the fingers and skin constrict intermittently in reaction to cold weather or emotional strain. This regulatory process is intended to avoid more heat loss and uphold central body temperature. Raynaud’s phenomenon can be classified into two types, either primary or secondary Raynaud’s [1]. Primary Raynaud’s occurs without an associated underlying condition, whereas secondary Raynaud’s is connected to various causes. Secondary Raynaud’s is primarily linked to conditions affecting connective tissues like scleroderma, systemic lupus erythematosus, Sjogren syndrome, and antiphospholipid syndrome [2]. Additional factors that play a role are the inclusion of specific medications such as antimigraine drugs, interferon alpha and beta, cyclosporine, nonselective beta-blockers, contact with polyvinyl chloride, cold work injuries, work with ammunition, Parvovirus B19, cytomegalovirus, hepatitis B, and hepatitis C [3]. Less frequent causes encompass fibromyalgia, polycythemia, arteriovenous fistula, myalgic encephalitis, and malignancies. In individuals over 60 years, obstructive vascular diseases, such as thromboangiitis obliterans, microemboli, diabetic angiopathy, and atherosclerosis, are frequent causes as well [3]. Other possible causes of Raynaud's phenomenon include conditions with comparable symptoms like external compression of blood vessels, complex regional pain syndrome, erythromelalgia, acute idiopathic blue finger (paroxysmal finger hematoma), acrocyanosis, occlusive peripheral vascular disease, peripheral neuropathy, and extreme cold sensitivity [3].

Nailfold capillary microscopy is utilized for diagnostic purposes to distinguish between primary and secondary Raynaud's phenomenon. This method evaluates changes in small blood vessels and structural characteristics in peripheral vessels such as changes in design, size of capillaries, density, presence of bleeding, and lack of blood flow areas. In terms of epidemiology, Raynaud's is more common in women, with a ratio of 9 females to 1 male, especially in younger age groups like teenagers and individuals in their twenties [4]. From a clinical standpoint, Raynaud’s usually impacts the outermost parts of the body, predominantly the fingers. An episode of Raynaud’s is sudden and results in frigid fingers and the appearance of either "white" areas (pallor) or "blue" areas (cyanosis) on the skin, lasting around 20 minutes. The onset of episodes commonly begins with one finger and then extends in a balanced manner to other fingers on both hands, typically leaving the thumb unaffected. The thumb being affected could signal secondary Raynaud’s [3]. Patients also frequently feel symptoms like tingling, numbness, or pain in their fingers during episodes. In serious instances, particularly in secondary Raynaud's phenomenon, digital ulcers or gangrene can occur as a result of extended vasoconstriction and resulting tissue ischemia. During a Raynaud’s episode, livedo reticularis can also occur, which features a purplish mottling or a reticular pattern caused by blood vessel clots [3]. This can be reversed by warming up but can become permanent due to secondary factors like antiphospholipid syndrome, vasculitis, cold agglutinin disease, or peripheral vascular disease. Here, we present an extremely rare case of primary Raynaud’s affecting the tongue.

## Case presentation

**Figure 1:**
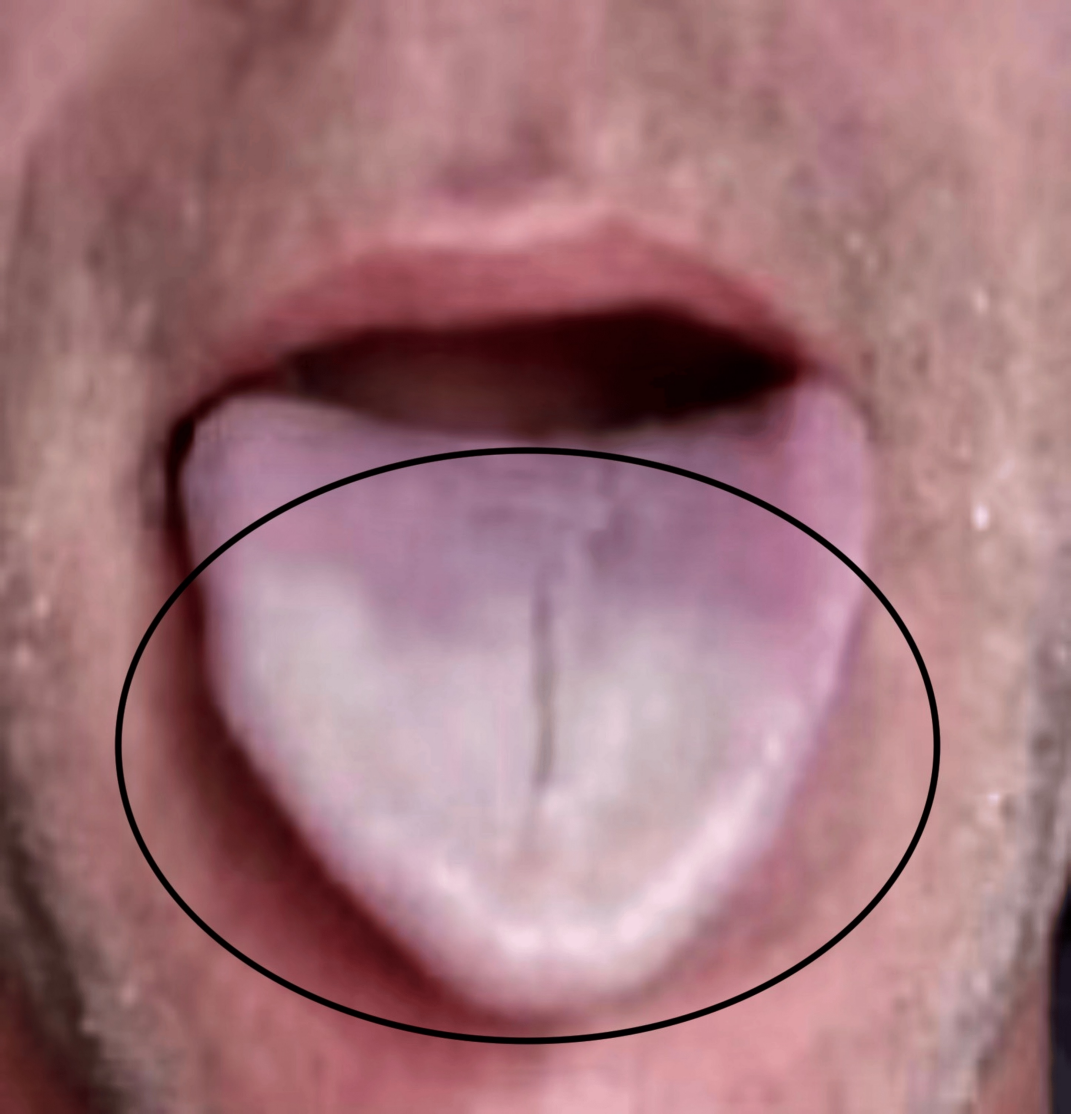
Clinical presentation of primary Raynaud's phenomenon affecting the tongue

A 51-year-old male presented to the clinic with complaints of recurrent episodes involving his distal one-third of the tongue becoming painful and turning white (Figure 1). These episodes, lasting between 10 to 30 seconds, had occurred 1-5 times annually over the past 3 years and had gradually increased in duration and affected a larger area of the tongue. The most recent episode lasted one to two minutes. The episodes were often preceded by numbness, tingling, and pain at the tongue tip. These symptoms occurred only when the tongue was white, gradually resolving as it reperfused. He reported no associated taste changes, dysphagia, globus sensation, or voice changes.

The physical examination revealed normal vitals: heart rate of 87, blood pressure of 126/86, and BMI of 24.15. The review of systems was notable for occasional fatigue, intermittent red blotching of cheeks bilaterally, multiple facial traumas including dog bites, occasional tongue biting, loud noise exposure at work, poor dentition due to past drug use, multiple root canals, palpitations, murmur, recent constipation, and back pain. The patient's past medical history included Mediterranean anemia, hyperlipidemia, gastroesophageal reflux disease (GERD), sciatica, torn right meniscus, torn rotator cuff, anxiety, and depression. He had a significant history of substance abuse, starting marijuana and alcohol use at age 10 and cocaine at age 12, but had been clean for the past 6.5 years. He also had a 25-year history of smoking one pack per day, having quit in July 2023. His family history included scleroderma in his mother; fibromyalgia and breast cancer in his sister; coronary artery disease and colon cancer in his father; and Mediterranean anemia in his mother, grandmother, and grandfather

Given the presentation and history, the patient was referred to rheumatology for further evaluation. The rheumatologic evaluation included tests for ANA, ENA, scl70, ACA, and dsDNA, all of which were negative, ruling out classic connective tissue diseases, including scleroderma. After the rheumatology assessment, the patient followed up with cardiology. The cardiology findings included normal left ventricular systolic function with an ejection fraction of 61%, normal-sized left ventricle cavity with normal global wall motion and diastolic filling pattern, normal-sized right atrial and right ventricle cavities with normal right ventricular function, and structurally normal trileaflet aortic valve with no regurgitation. The aortic valve peak gradient was 16 mmHg, the mean gradient was 9 mmHg, and the aortic valve area indexed to body surface area was 1.27 cm²/m². Additionally, the mitral valve was structurally normal with trace regurgitation, the tricuspid valve had mild regurgitation, and the pulmonic valve had trace regurgitation. There was no evidence of significant pericardial effusion, the aortic root was normal, the pulmonary artery was normal, and the inferior vena cava was normal with respiratory variation.

## Discussion

Raynaud’s phenomenon is a complex condition involving reduced blood flow, narrowing of blood vessels, nerve-related reactions, and inflammation and immune system activation. The somatosensory system controls temperature perception by activating afferent nerve fibers in response to cold temperatures, which in turn stimulates A-delta and unmyelinated C-fibers. This stimulation activates the TRPM8 cold receptor, which detects changes in cold temperatures and causes skin blood vessel constriction. The sympathetic nervous system also plays a role in secondary Raynaud's by releasing neuropeptides and norepinephrine, leading to vasoconstriction due to endothelin-1 released by endothelial cells [3].

Primary Raynaud's is characterized by heightened sensitivity of alpha-2 adrenergic receptors in digital and cutaneous vessels, causing vasoconstriction when exposed to cold and emotional stress. The distal arterial smooth muscles of the digits are affected by the sympathetic nervous system through receptors found in that area. Research has indicated that the use of alpha-2 adrenergic receptor inhibitors can lessen the intensity of cold-induced episodes. In secondary Raynaud's, the original disease disturbs typical blood vessel responsiveness, resulting in endothelial dysfunction and subsequent narrowing of blood vessels and tissue damage. An instance of this is when systemic scleroderma leads to vascular fibrosis, causing endothelial dysfunction and ischemia [5].

The goal of treating Raynaud's is to lessen the frequency and intensity of episodes and avoid tissue damage from lack of blood flow [6]. Conservative treatment involves lifestyle changes like staying warm, avoiding cold, managing stress, quitting smoking, and avoiding stimulants. If these steps are not enough, vasodilators are utilized for pharmacologic treatment. Initial therapy consists of dihydropyridine calcium channel blockers (DHP CCBs) like amlodipine and nifedipine, which are initiated with small doses and increased slowly over time. Possible adverse reactions consist of peripheral swelling, rapid heartbeat, reflexively increased heart rate, lightheadedness, and migraines. Some reasons to avoid using this treatment are low blood pressure, swelling in the limbs, chest pain, heart attack, aortic valve narrowing, thickened heart muscles, and high blood pressure in the lungs. CCBs are not recommended during pregnancy unless digital ischemia is present [6]. In patients who do not have a positive reaction to DHP CCBs, phosphodiesterase (PDE) inhibitors, such as sildenafil, can be included or used alone. Sildenafil is initiated with a low dose and adjusted gradually within four to six weeks. Additional options consist of topical nitrates, for example, nitroglycerin cream, which may result in systemic side effects such as low blood pressure, lightheadedness, and headaches. Other potential treatment choices for cases that do not respond to treatment include losartan, fluoxetine, and prazosin. Serious situations involving tissue ischemia may need intravenous prostaglandin infusions (iloprost, epoprostenol, treprostinil, alprostadil) with potent vasodilatory and antiplatelet aggregation properties. Bosentan, a medication that blocks the endothelin-1 receptor, is prescribed for recurring ulcers on the fingers in connective tissue disorders such as systemic sclerosis [3]. In severe, refractory cases, surgical sympathectomy is considered as a treatment option, involving the removal of sympathetic nerves responsible for vasoconstriction. For individuals who cannot tolerate low-dose aspirin, clopidogrel or dipyridamole are recommended as alternatives in cases of secondary Raynaud phenomenon with ischemic ulceration [7].

Primary Raynaud's may improve on its own while secondary Raynaud's phenomenon usually does not show improvement. Pregnancy may benefit Raynaud's by increasing blood flow and oxygenation in the extremities through higher red blood cell mass and plasma volume. Severe Raynaud's can result in tissue ischemia, which may lead to necrosis and possible amputation of the affected area [7].

## Conclusions

Raynaud’s phenomenon represents a dynamic interplay of vascular, neurogenic, and immune factors leading to episodic vasoconstriction primarily in the extremities. This condition, split into primary and secondary forms, poses varied etiologies ranging from isolated vascular reactivity to associations with systemic diseases and external factors. Effective management hinges on a tailored approach, prioritizing conservative measures and escalating to pharmacological or surgical interventions as necessary. The nuanced understanding of its pathophysiology is critical for timely diagnosis and tailored therapeutic strategies, which are essential to mitigate its impact and enhance the quality of life for affected individuals.
